# Early Post-discharge Telephone Follow-Up in a Psychiatric ED: A Quality Improvement Initiative to Strengthen Care Transitions and Patient Engagement

**DOI:** 10.7759/cureus.112101

**Published:** 2026-07-05

**Authors:** Bhargav Patel, Jay Gopal, Christian Little, Urja Shah, Erin Dexter

**Affiliations:** 1 Department of Psychiatry and Human Behavior, Brown University, Providence, USA; 2 Medical School, Brown University, Providence, USA; 3 Department of Psychiatry, Wellstar Medical College of Georgia Health Medical Center, Augusta, USA; 4 Department of Psychiatry, Icahn School of Medicine at Mount Sinai, New York, USA; 5 Department of Psychiatry, Medical College of Georgia, Augusta University, Augusta, USA

**Keywords:** care coordination, discharge transitions, follow-up calls, medication adherence, patient satisfaction, psychiatric emergency department, quality improvement

## Abstract

Background

Psychiatric EDs manage high volumes of patients who often face barriers to medication access, limited understanding of discharge instructions, and difficulty securing timely outpatient follow-up. Structured early outreach has shown promise in improving these transitions. This quality improvement initiative evaluated a 72-hour nurse-led telephone follow-up program for patients discharged from a psychiatric service within an ED.

Methods

All patients discharged over the study period were eligible for a structured telephone call within 72 hours. The call assessed medication pickup, medication adherence, follow-up appointment status, transportation needs, and satisfaction with both emergency care and the follow-up call. Diagnoses were extracted from clinician documentation. Demographic characteristics and patient-reported needs were summarized. Outcomes included call reach rate, patient-reported understanding of discharge instructions, medication-related behaviors, and follow-up completion.

Results

A total of 69 patients met the inclusion criteria, with a mean age of 35.3 years. The most common diagnoses were major depressive disorder with suicidal ideation, schizophrenia, and bipolar disorder. The nurse navigator successfully contacted 37.7% of discharged patients. Among reached patients, half reported taking their medications, and over two-thirds had a scheduled follow-up appointment. Transportation barriers were uncommon. Patient satisfaction with psychiatric emergency care averaged 4.45 on a five-point scale, and satisfaction with the follow-up call averaged 4.95. Patients commonly sought clarification regarding medications, follow-up appointments, and transportation resources.

Conclusion

Early nurse navigator-led telephone follow-up after discharge from psychiatric emergency care demonstrated strong patient engagement and high satisfaction. The calls created opportunities to reinforce discharge instructions, support medication use, and assist with follow-up planning. These findings suggest that early nurse-led contact can highlight actionable barriers and support safer care transitions following psychiatric emergency discharge.

## Introduction

Hospital discharge is a vulnerable transition in the care continuum, particularly for patients leaving emergency and psychiatric settings, where unresolved questions and limited support can affect safety and subsequent healthcare use [1,2]. Studies have shown that many patients leave the hospital or ED with an incomplete understanding of follow-up plans, medication changes, and warning signs, which can influence their ability to manage care at home [3,4]. These communication gaps have been associated with unanswered concerns, missed appointments, medication nonadherence, and preventable return visits to acute care settings [3-6]. Psychiatric populations face additional challenges related to complex treatment needs, community coordination, and barriers to outpatient engagement, which place them at elevated risk for early readmission [7,8].

Structured post-discharge communication has been proposed as a strategy to address these gaps. Post-discharge telephone follow-up refers to structured telephone contact initiated by a clinician after hospital discharge to reconcile medications, reinforce discharge instructions, identify emerging complications, and confirm follow-up appointments; such contact is generally considered early when completed within 24-72 hours of discharge, with 48-72 hours being the most commonly applied window [9-13]. Systematic reviews of discharge communication interventions have reported improvements in patient satisfaction, self-management, and understanding of treatment plans [9]. Early work demonstrated that most patients contacted by telephone within two to three days of discharge had questions about self-care that were not addressed during hospitalization [10]. Subsequent studies have linked telephone follow-up with greater clarity of medication instructions and more reliable completion of follow-up appointments [11]. Large cohort studies have also reported reductions in 30-day readmission among patients who received a timely discharge call, including a study of more than 30,000 patients that found a significant reduction in readmission risk when outreach was completed within 14 days [12]. Integrated health systems implementing nurse-led scripted follow-up programs within 72 hours have similarly reported lower seven-day and 30-day readmission rates among patients successfully contacted [13].

Automated telephone programs extend these efforts by offering structured outreach at scale and have demonstrated high reach across age groups, including older adults [14]. In automated models, programs typically use interactive voice response (IVR) technology to place pre-scheduled calls that deliver a standardized, branching script: patients confirm their identity; respond to prompts about symptoms, medication adherence, and appointment status using the keypad or voice; and are automatically escalated to a live nurse or care coordinator when responses indicate a clinical concern or unmet need. In nurse-led models, a dedicated nurse navigator places a direct call to each discharged patient and works through a similar script, confirming patient identity, reviewing discharge instructions and medications, screening for new or worsening symptoms, verifying that follow-up appointments are scheduled, and answering patient questions. Work in older adults has shown strong engagement with automated calls and frequent questions regarding follow-up planning and appointment scheduling [14]. Transitional care programs that incorporate multiple follow-up calls have been associated with higher completion of recommended post-discharge activities, suggesting that call frequency and call quality influence effectiveness [15]. In psychiatric settings, multistage evaluations emphasize the importance of consistent communication, patient and family education, team-based coordination, and clear processes for addressing emerging concerns during the transition period [7]. Beyond clinical outcomes, the transition from hospital to home is also a key determinant of patient satisfaction. Prior work has shown that patients report continued, often unmet, education needs after discharge and that the perceived importance of discharge topics increases once patients are home, indicating greater readiness to engage with follow-up communication during this period [10].

Given the continued burden of psychiatric ED readmissions and the importance of reliable discharge transitions, targeted evaluations of early post-discharge telephone outreach are needed. The aim of this quality improvement initiative was to examine a 72-hour nurse-led follow-up call program for patients discharged from a psychiatric ED, focusing on patient satisfaction, engagement with discharge instructions, and transitional care barriers such as medication pickup and follow-up scheduling. As a single-site quality improvement effort, this work was designed to assess feasibility and acceptability rather than to test hypotheses or measure readmission outcomes directly.

## Materials and methods

This quality improvement initiative was conducted in the psychiatric ED at Augusta University Hospital, Augusta, USA, between July 1, 2022, and October 10, 2022, to evaluate the feasibility and utility of structured post-discharge outreach. No patient data were collected specifically for research purposes; all data were derived from routine clinical operations as part of an internal departmental quality improvement and quality assurance project that was written up afterward. The project was determined not to require Institutional Review Board review based on its nature, as assessed using the University of Wisconsin-Madison QI/Program Evaluation Self-Certification Tool, which returned the determination that the project constituted quality improvement and program evaluation and did not meet the definition of research under 45 CFR 46.102(l); IRB review was therefore not required. Because this was a single-site quality improvement initiative rather than a hypothesis-testing study, no a priori sample size calculation was performed; instead, all consecutive patients discharged during the implementation period were screened, yielding a convenience sample of 69 eligible patients. Inclusion criteria were (1) discharge from the psychiatric ED during the implementation period and (2) documented age, gender, discharge diagnosis, and contact information in the clinical record. Exclusion criteria were (1) transfer to inpatient psychiatric admission rather than community discharge and (2) missing demographic, diagnostic, or contact information that precluded structured outreach.

Diagnoses were abstracted from clinician entries and reflected the working diagnoses assigned by the evaluating psychiatric provider during the emergency encounter, applying standard Diagnostic and Statistical Manual of Mental Disorders, Fifth Edition, Text Revision (DSM-5-TR) criteria as part of routine clinical assessment. No separate structured diagnostic instrument was administered for this initiative. In several cases, multiple diagnoses were identified. Diagnostic categories included schizophrenia, bipolar disorder, major depressive disorder with suicidal ideation, major depressive disorder, generalized anxiety disorder, and other psychiatric conditions.

A nurse navigator attempted a structured telephone call for each discharged patient within 72 hours of discharge. Up to three call attempts were made per patient over the course of a single day before the patient was recorded as unreachable. Call duration was not recorded. The call script included questions regarding medication pickup, medication adherence, follow-up appointment status, transportation availability, and outstanding questions. The follow-up appointment item referred to a future scheduled outpatient visit after the emergency encounter. The script was reviewed for clarity and content by the head ED psychiatrist before use; no formal psychometric validation was performed, consistent with its role as an operational quality improvement tool. All call responses were recorded and abstracted by the single nurse navigator who conducted the calls; therefore, inter-rater reliability could not be assessed. Patients were also asked to rate their satisfaction with ED care and with the follow-up call on a five-point Likert scale. Outcomes were defined as follows. Reach was defined as a completed call in which the patient answered and engaged with the script. Medication pickup was defined as patient-reported collection of discharge prescriptions from a pharmacy. Medication adherence was defined as patient-reported use of prescribed medications. The follow-up item referred to a future outpatient visit scheduled after the emergency encounter, either already arranged at discharge or confirmed during the call. Transportation need was defined as patient-reported availability of reliable transportation to follow-up care. Satisfaction was defined as the patient’s rating of ED care and of the follow-up call. Categorical items were coded as one for “yes” and two for “no,” and satisfaction ratings ranged from one (lowest) to five (highest). Only patients who answered the call were included in analyses of medication behavior, follow-up participation, transportation needs, and satisfaction.

Descriptive statistics were used to summarize all variables. Continuous variables were characterized using means, SD, medians, and ranges, while categorical variables were summarized using counts and percentages. Given the small convenience sample and the descriptive quality improvement design, no inferential or comparative testing between reached and unreached patients was performed; reported proportions are accompanied by 95% CIs calculated using the Wilson method to convey precision. All analyses were performed using Python version 3.11 (Python Software Foundation, 2023), with the pandas (v2.0.3), NumPy (v1.24.3), and Matplotlib (v3.7.1) libraries used for data handling, descriptive statistics, and figure generation.

## Results

A total of 69 patients met the inclusion criteria. The mean age was 35.35 years, the median age was 30 years, the SD was 14.44 years, and ages ranged from 18 to 73 years. Gender distribution was nearly even, with 35 patients (50.7%) identified as female and 34 patients (49.3%) identified as male (Table 1).

**Table 1:** Demographic and clinical characteristics of the analytic cohort (N = 69). Demographic and clinical characteristics of all 69 patients are presented, including age distribution (mean, median, SD, and range), gender distribution, recorded follow-up location, and frequencies of each psychiatric diagnostic category based on parsed diagnosis codes. Individual patients may have had multiple diagnoses; therefore, diagnosis percentages may add to more than 100%. Continuous variables are presented as mean ± SD, and categorical variables are presented as n (%). No inferential statistical tests were applied; values are descriptive only. Locations refer to the outpatient behavioral health program or facility recorded as the patient’s follow-up destination after discharge. American Works and Serenity are state-funded community mental health centers. Stoney refers to Augusta University Hospital’s psychiatry outpatient clinic. “Other or unknown” denotes patients referred elsewhere or patients for whom a specific program was not documented, which was common because this field was not required during routine psychiatric emergency care. MDD: Major depressive disorder; SI: Suicidal ideation; GAD: Generalized anxiety disorder.

Category	Variable	Value
Demographics	Age	35.35 ± 14.44 years
	Female	35 (50.72%)
	Male	34 (49.28%)
Follow-up location	American Works	4 (5.80%)
	Stoney	4 (5.80%)
	Serenity	7 (10.14%)
	Other or unknown	54 (78.26%)
Diagnoses	MDD with SI	29 (42.03%)
	Bipolar disorder	12 (17.39%)
	Schizophrenia	12 (17.39%)
	Other	9 (13.04%)
	GAD	5 (7.25%)
	MDD	3 (4.35%)

Diagnostic patterns reflected a heterogeneous but high-acuity psychiatric cohort. Using the 69-patient cohort denominator, major depressive disorder with suicidal ideation accounted for 29 instances (42.0%), schizophrenia and bipolar disorder were each coded 12 times (17.4%), “other” diagnoses nine times (13.0%), generalized anxiety disorder five times (7.3%), and major depressive disorder without suicidal ideation three times (4.4%) (Table 1). The 70 coded diagnoses exceeded the 69 unique patients because one patient had two diagnoses documented (Table 2).

**Figure 1:**
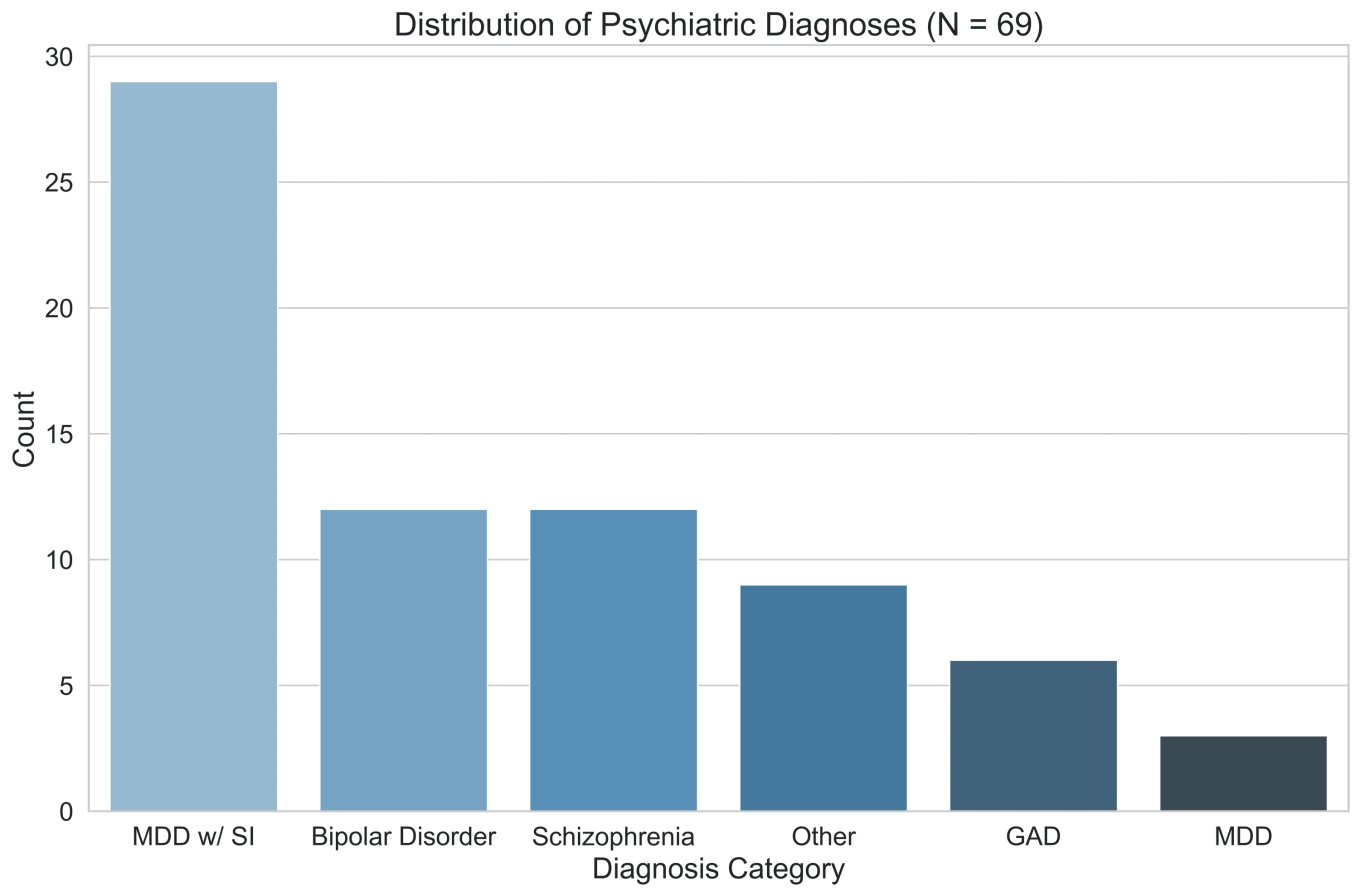
Distribution of psychiatric diagnoses among discharged patients (N = 69). Distribution of coded psychiatric diagnoses among the 69 patients in the analytic cohort. Diagnoses were extracted from clinician documentation, and each diagnosis was counted independently. Major depressive disorder with suicidal ideation, schizophrenia, bipolar disorder, generalized anxiety disorder, major depressive disorder, and other conditions are displayed as separate categories. Values represent counts and percentages, n (%), using the total number of coded diagnoses (n = 70) as the denominator. The total number of coded diagnoses exceeds the number of patients because one patient had two documented diagnoses. No inferential statistical tests were applied. MDD: Major depressive disorder; SI: Suicidal ideation; GAD: Generalized anxiety disorder.

**Table 2:** Distribution of coded psychiatric diagnoses in the full cohort. Coded psychiatric diagnoses across the full cohort are shown. The 70 coded diagnoses exceed the 69 patients because one patient had two documented diagnoses: MDD and GAD. Percentages were calculated using the total number of coded diagnoses as the denominator. MDD: Major depressive disorder; SI: Suicidal ideation; GAD: Generalized anxiety disorder.

Diagnosis	Revised n (%)
MDD with SI	29 (41.4)
Bipolar disorder	12 (17.1)
Schizophrenia	12 (17.1)
Other	9 (12.9)
GAD	5 (7.1)
MDD	3 (4.3)
Total coded diagnoses	70

**Figure 2:**
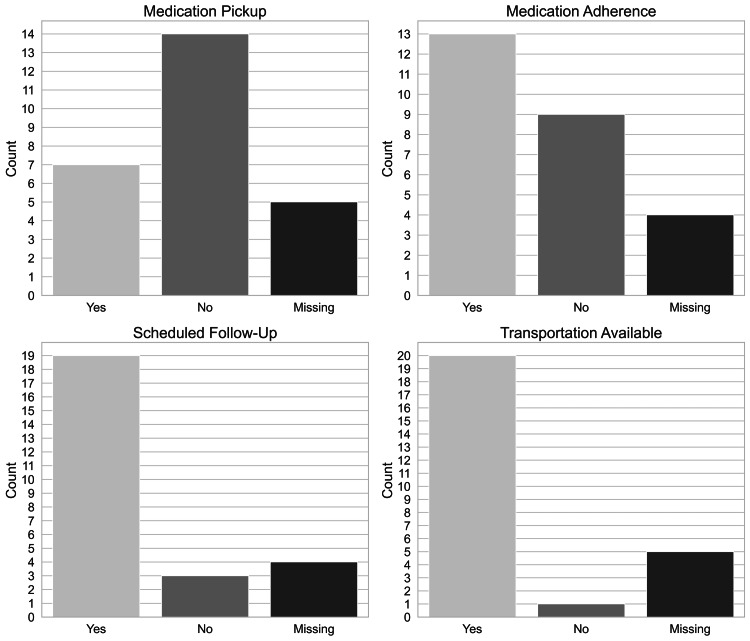
Post-discharge follow-up call responses for medication, follow-up, and transportation (n = 26). Responses to medication pickup, medication adherence, scheduled follow-up appointments, and transportation availability among the 26 patients who answered the 72-hour post-discharge call. For each item, the figure displays the proportion of contacted patients with recorded “yes,” “no,” and missing responses. Values represent n (%) of contacted patients (n = 26). Categories within each item sum to 100%. No inferential statistical tests were applied.

The nurse navigator successfully contacted 26 of 69 discharged patients (37.7%, 95% CI 27.2% to 49.5%) within 72 hours. Among the 35 female patients, 16 (45.7%) answered the call, whereas 10 of 34 male patients (29.4%) answered. All subsequent analyses of medication behavior, follow-up status, transportation, and satisfaction were restricted to these 26 contacted patients (Figure 2). For the medication pickup item, seven of the 26 patients (26.9%, 95% CI 13.7% to 46.1%) reported having filled their prescriptions, 14 (53.8%) reported that medications had not yet been picked up, and five (19.2%) had no response recorded. For the item on taking medications, 13 patients (50.0%, 95% CI 32.1% to 67.9%) reported taking medications as prescribed, nine (34.6%) reported not taking them, and four (15.4%) had missing responses. The higher proportion reporting adherence than reporting pharmacy pickup likely reflects patients already taking previously prescribed medications, those given starter supplies at discharge, and the slightly different reference frames of the two questions. These distributions are summarized for contacted patients in Figure 2 and Table 3.

**Table 3:** Post-discharge call responses for medication, follow-up, and transportation among contacted patients (n = 26). Self-reported responses to each call item among the 26 patients who answered the 72-hour post-discharge call. Values are n (%) of contacted patients, and categories within each item sum to 100%. “Missing” denotes patients who answered the call but had no response recorded for that item. No inferential statistical tests were applied; values are descriptive only.

Item	Yes, n (%)	No, n (%)	Missing, n (%)
Medication pickup	7 (26.9)	14 (53.8)	5 (19.2)
Taking medications as prescribed	13 (50.0)	9 (34.6)	4 (15.4)
Scheduled follow-up appointment	19 (73.1)	3 (11.5)	4 (15.4)
Transportation available	20 (76.9)	1 (3.8)	5 (19.2)

Follow-up appointment status was more completely documented. Among the 26 contacted patients, 19 (73.1%, 95% CI 53.9% to 86.3%) had a scheduled future follow-up visit recorded, three (11.5%) reported no scheduled appointment, and four (15.4%) had missing data for this item (Figure 2). Transportation status was coded for most contacted patients: 20 (76.9%, 95% CI 57.9% to 89.0%) had transportation recorded as available, one (3.8%) had transportation recorded as not available, and five (19.2%) had no transportation status documented (Table 3). These values reflect the subset of patients who both answered the call and had non-missing entries in the corresponding fields.

**Figure 3:**
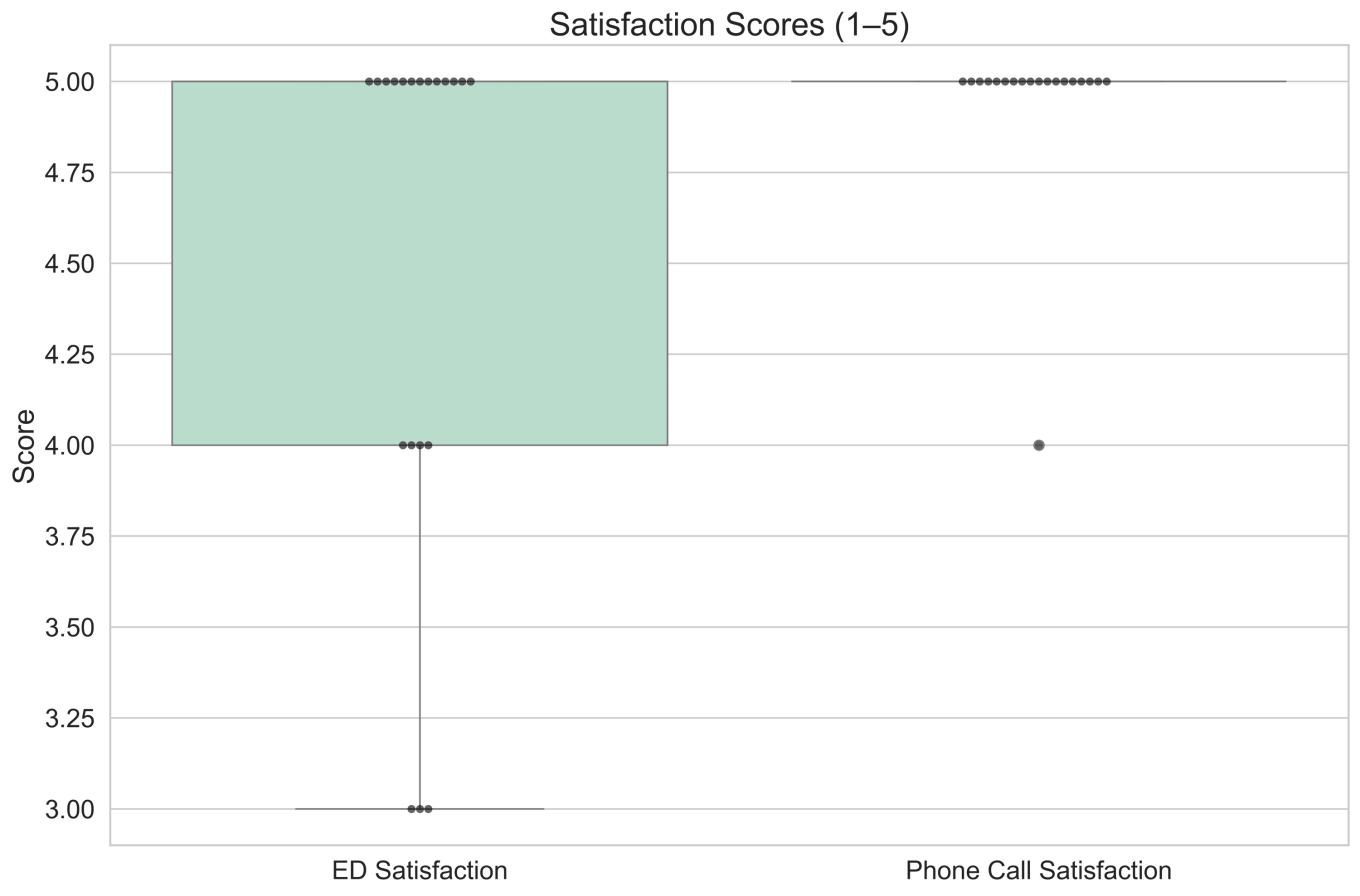
Distribution of satisfaction scores for ED care and the follow-up call (n = 26) Distribution of patient satisfaction scores for ED care and the 72-hour follow-up call among contacted patients with non-missing ratings. Satisfaction was rated on a five-point Likert scale, where 1 indicated the lowest satisfaction and 5 indicated the highest satisfaction. The figure illustrates the spread and central tendency of scores for both facets of care. Box plots show the median (center line), IQR (box), and full range of satisfaction scores, with whiskers extending to the minimum and maximum and individual points denoting outliers. Ratings were measured on a five-point Likert scale (n = 20 for ED satisfaction; n = 19 for follow-up call satisfaction). No inferential statistical tests were applied.

Satisfaction scores were available only for contacted patients who provided ratings. For satisfaction with ED care, 20 of the 26 contacted patients had non-missing responses. Among these, the mean satisfaction score was 4.45 on a five-point scale, with scores ranging from three to five. Three patients (15.0% of those with scores) selected a rating of three, four patients (20.0%) selected four, and 13 patients (65.0%) selected five. For satisfaction with the follow-up call, 19 contacted patients had recorded scores. The mean satisfaction score for the call was 4.95, with scores ranging from four to five (Table 4). One patient (5.3% of those with scores) selected four, and 18 patients (94.7%) selected five (Figure 3). Patients with missing satisfaction ratings were included in the contacted denominator when describing reach and behavioral outcomes but were excluded from the calculation of satisfaction means and distributions.

**Table 4:** Distribution of patient satisfaction scores for ED care and the follow-up call (n = 26). Satisfaction ratings on a five-point Likert scale are shown for contacted patients with non-missing responses, where 1 indicated the lowest satisfaction and 5 indicated the highest satisfaction. Denominators differ because six patients had no ED care rating and seven had no follow-up call rating. Percentages were calculated within each column. No inferential statistical tests were applied; values are descriptive only.

Score	ED care, N (%) (n = 20)	Follow-up call, N (%) (n = 19)
3	3 (15.0)	0 (0.0)
4	4 (20.0)	1 (5.3)
5	13 (65.0)	18 (94.7)
Mean (range)	4.45 (3 to 5)	4.95 (4 to 5)

Within the contacted group, diagnostic patterns remained similar to the full cohort (Table 5). When diagnoses for contacted patients were parsed and counted, major depressive disorder with suicidal ideation appeared 10 times, bipolar disorder seven times, schizophrenia and "other" diagnoses three times each, and generalized anxiety disorder and major depressive disorder without suicidality twice each. The 27 coded diagnoses exceed the 26 contacted patients because one contacted patient had two diagnoses documented. The follow-up sample therefore retained representation from each diagnostic category present in the broader discharged population.

**Table 5:** Distribution of psychiatric diagnoses among contacted patients (n = 26). Coded psychiatric diagnoses among the 26 patients who answered the post-discharge call are shown, with each diagnosis counted independently. The 27 coded diagnoses exceed the 26 patients because one contacted patient had two documented diagnoses: MDD and GAD. The contacted sample retained representation from every diagnostic category present in the full cohort. No inferential statistical tests were applied; values are descriptive only. MDD: Major depressive disorder; SI: Suicidal ideation; GAD: Generalized anxiety disorder.

Diagnosis	n
MDD with SI	10
Bipolar disorder	7
Schizophrenia	3
Other	3
GAD	2
MDD	2
Total coded diagnoses	27

## Discussion

In our initiative, a nurse-led telephone outreach within seventy-two hours of discharge from a psychiatric ED enabled targeted engagement around medication pickup, appointment scheduling, and transition-related questions. By reaching one in three discharged patients and achieving strong satisfaction ratings, the program demonstrates potential as a practical transitional care component in a psychiatric emergency context.

This work corroborates and extends prior research examining post-discharge telephone follow-up in psychiatric and emergency settings. For example, in a controlled study of 436 patients treated for attempted suicide, very early telephone follow-up at 8, 30, and 60 days was associated with reduced repeat attempts [16]. That study focused on a higher-risk inpatient population and assessed long-term behavioral outcomes, whereas our effort examines the more immediate post-discharge timeframe in a psychiatric ED. The present study emphasizes engagement and transitional metrics, such as medication pickup and follow-up scheduling, rather than longer-term outcomes. Another recent large retrospective cohort study in children and adolescents found that continuity of clinician from emergency presentation to community follow-up increased the odds of post-discharge outpatient follow-up by more than threefold [17]. While their work emphasizes continuity of care, ours emphasizes early outreach to improve the transition itself, a relatively nascent area of research.

In the broader hospital discharge literature, navigator calls, scripted telephone contacts, and structured outreach have been linked to improved medication clarity, better understanding of follow-up, and, in some settings, reduced readmissions [18]. Our findings thus add to a growing body of evidence that simple telephone interventions may improve patient outcomes and satisfaction [19].

The exceptionally high satisfaction with the follow-up calls, reflected by a mean rating of 4.95 out of 5, underscores the degree to which patients valued a brief, personal, and supportive touchpoint during a vulnerable transition period.

From a patient-centered perspective, these calls may serve functions that extend beyond logistical clarification: they provide validation, continuity, and a sense of being remembered after an acute crisis encounter [20]. Psychiatric emergency presentations often leave patients feeling destabilized, disconnected, or unsure about next steps; thus, even a short, structured call may restore a sense of safety and belonging, consistent with caring contact interventions that reduce isolation and enhance connectedness following a psychiatric crisis [20]. The overwhelmingly positive response suggests that patients perceived the outreach as both meaningful and reassuring, reinforcing the broader literature indicating that human connection during transitions of care can support engagement, understanding, and trust [19]. This finding highlights that the value of follow-up calls lies not only in reinforcing instructions but also in supporting the emotional and relational dimensions of care transitions. At the same time, high satisfaction should not be read as evidence of clinical effectiveness. Satisfaction measures patient experience, which correlates inconsistently with objective outcomes such as adherence, readmission, and symptom improvement, and in some studies tracks weakly or inversely with them [21]. The scores here indicate that patients valued the contact and found it acceptable, not that the calls altered their clinical trajectory, which this design cannot establish.

The present study’s significance lies in tailoring the intervention to a psychiatric ED environment, where patients are discharged after a crisis presentation and often face multiple barriers such as symptom severity, social instability, and fragmented outpatient follow-up [8]. The 37.7% reach rate may appear modest, but it may be expected for this high-vulnerability group, given that fewer than half of these patients successfully transition to outpatient care at all [22]. Several factors likely contributed to the limited reach. Psychiatric emergency patients frequently have unstable housing, disconnected or changing phone numbers, and discharge to settings other than a personal residence, all of which reduce telephone connectivity [12]. Symptom severity, ongoing crisis, and ambivalence about continued care may also lower the likelihood of answering an unfamiliar number shortly after discharge. Prior work on telephone outreach has found that reach is difficult to predict from routine sociodemographic data alone, which limits the ability to target the hardest-to-reach patients in advance [23]. Female patients answered more often than male patients (45.7% versus 29.4%), consistent with broader patterns of greater engagement with health services among women, though the small numbers preclude firm conclusions. The fact that many patients reported medication non-pickup or lack of confirmed follow-up underscores the value of timely contact. Furthermore, the high satisfaction scores suggest that patients welcomed the contact, which may foster engagement beyond the call itself.

A few limitations are important to consider. First, the absence of a control or comparison group limits causal inference about how much the outreach improved outcomes compared with usual care, and the small convenience sample precluded meaningful inferential comparison between reached and unreached patients. Because satisfaction and behavioral data were available only for patients who answered the call, these results are subject to selection bias: patients who answered may differ systematically from those who did not in social support, symptom severity, or stability of contact information, which likely inflates the favorable satisfaction and engagement figures. All call data were also recorded by a single nurse navigator, so inter-rater reliability could not be assessed. Furthermore, outcomes such as medication adherence and follow-up scheduling relied on patient self-report within the call and were not independently verified by pharmacy records or outpatient appointment attendance data; self-report is also subject to social desirability bias. One internal inconsistency illustrates this: more patients reported taking medications (50.0%) than reported picking them up (26.9%), most plausibly because some were continuing previously prescribed medications or received supplies at discharge, and because the two questions used slightly different reference frames. We also did not assess downstream clinical endpoints such as seven-day or 30-day readmission, emergency re-presentation, or clinical symptom trajectories, which were beyond the scope of this quality improvement initiative. Additionally, future studies should consider collecting details such as call duration. Follow-up studies may consider validating our findings in larger patient cohorts with verification of self-reports. Finally, the intervention focused on a single call within 72 hours; future work should assess the impact of multiple calls or longer outreach windows.

Several extensions of this work warrant investigation. First, larger multi-site studies with control or historical comparison groups are needed to establish causal effects of early nurse-led outreach on hard endpoints, including seven-day and 30-day psychiatric ED re-presentation, inpatient readmission, and outpatient follow-up attendance. Second, self-reported behaviors should be validated against objective sources such as pharmacy fill records and outpatient appointment attendance data. Third, future protocols should test the incremental value of multiple call attempts, varied call windows, and mixed outreach modalities, such as text messaging and automated voice systems, on contact rates, particularly among demographic subgroups with lower reach in the present cohort. Fourth, integrating the nurse navigator role with peer support specialists or community mental health liaisons may improve engagement for patients with severe mental illness. Finally, prospective evaluation of cost-effectiveness and staffing models will be needed to support broader implementation across psychiatric emergency settings.

In summary, our quality improvement initiative demonstrates that early nurse-led telephone follow-up after psychiatric emergency discharge is feasible, acceptable to patients, and offers actionable insights into transitional care barriers. Several practical implications follow. The intervention requires only a single nurse navigator working from a brief script, so departments can implement it without new technology or major staffing changes. The calls surface concrete, actionable gaps in real time, such as unfilled prescriptions and unconfirmed appointments, which allows staff to intervene during the highest-risk window rather than waiting for a missed visit or return to the ED. The low reach rate also carries an operational lesson: contact information should be verified before discharge, and call attempts should be spread across different times of day, with text or automated outreach considered as a backstop for patients who do not answer. Because reach was lower among male patients, departments may want to track contact rates by subgroup and tailor outreach accordingly. Taken together, these points suggest that the program is a pragmatic and scalable strategy to bolster care transitions in a high-risk setting. Further evaluation is needed to test its impact on clinical outcomes and to optimize implementation for broader use.

## Conclusions

Early nurse-led telephone follow-up after discharge from a psychiatric ED was feasible and well received, providing a structured opportunity to address medication questions, clarify follow-up plans, and identify unresolved transition-related needs. Although reach was limited, patients who were contacted reported high satisfaction and frequently received additional guidance shortly after discharge. These findings support early post-discharge outreach as a pragmatic quality improvement strategy to strengthen care transitions in psychiatric emergency settings. Further work should evaluate methods to improve contact rates and assess downstream effects on outpatient engagement and acute care utilization.
